# Price differentials of tobacco products: A cross-sectional analysis of 79 countries from the six WHO regions

**DOI:** 10.18332/tid/142550

**Published:** 2021-10-15

**Authors:** Christina N. Kyriakos, Aulia Ahmad, Kiara Chang, Filippos T. Filippidis

**Affiliations:** 1 Public Health Policy Evaluation Unit, School of Public Health, Imperial College London, London, United Kingdom

**Keywords:** tobacco taxation, price differentials, tobacco products, smoking, WHO regions

## Abstract

**INTRODUCTION:**

Increased taxation is one of the most effective tobacco control measures. Price differentials across tobacco product types may undermine the effectiveness of taxation policies by providing the option to switch to cheaper products rather than to quit. The aim of this study was to use commercial data to compare prices and price differentials of both cigarette and non-cigarette products across countries from all geographical regions.

**METHODS:**

We analyzed 6920 price data points (i.e. product brands) from Euromonitor Passport 2016 for 12 types of tobacco products across 79 countries from the six WHO regions: Africa (n=5), Eastern Mediterranean (n=6), Europe (n=39), the Americas (n=15), South-East Asia (n=3), and Western Pacific (n=12). For each product and country, a price differential was computed as the percentage of minimum price to the median.

**RESULTS:**

Median cigarette prices (US$) were highest in Western Pacific countries (4.00; range: 0.80–16.20) and European countries (3.80; range: 0.80–14.00), but lowest in African countries (2.00; range: 0.80–2.20). The medians of cigarette price differentials were largest in the Eastern Mediterranean (48.33%) and African regions (50.00%), but smallest in Europe (82.35%). Pipe tobacco and fine-cut tobacco were generally less expensive than cigarettes while cigars were the most expensive. However, there were wide variations in prices and price differentials across regions and tobacco products.

**CONCLUSIONS:**

We found substantial variations in prices and price differentials between countries and world regions across tobacco products, likely reflecting differences in taxation policies and structures. Findings identify types of tobacco products in specific geographical regions where price differentials are highest, thereby highlighting areas where taxation policies need improvement, for example through implementing specific excise taxes.

## INTRODUCTION

Despite global progress in tobacco control, smoking remains the largest cause of preventable mortality, with 8 million deaths attributable to tobacco annually^1^. Increasing tobacco prices by raising taxes is considered the single most effective and cost-effective tobacco control measure and is the cornerstone of both the World Health Organization (WHO) Framework Convention on Tobacco Control (FCTC) and the MPOWER policy package which lays out six strategies for reducing the demand of tobacco products, including ‘R’ for ‘Raise taxes on tobacco’^2,3^. Higher cigarette prices have repeatedly been shown to reduce smoking initiation and tobacco consumption, and increase quitting^4,5^. A rise in taxation is most effective when it leads to increases in the price of tobacco products which are above inflation and income growth^6^.

As of 2020, only 40 countries worldwide have instituted taxation on cigarettes that are at least 75% of the retail price, with the majority of these countries being high-income^1^. Overall progress on WHO FCTC Article 6, which requires ratifying Parties to implement tax and price measures to reduce the demand for tobacco, has been slow^7^. The situation is further compounded by tobacco industry actions designed to minimize or offset the impact of tax policies. For example, tobacco companies may introduce cheaper products so that costs of tax increases are absorbed by the industry^8^. The resulting price differentials between premium and budget products may encourage users to switch to a cheaper product rather than to quit, although such behaviors may be influenced by multiple factors^9^. Availability of lower priced products has also been shown to be associated with reduced motivation to quit and cessation success^10^. These tobacco industry tactics likely attenuate the effectiveness of taxation policies, especially when tobacco taxation structures rely heavily on *ad valorem* taxes^11^.

While cigarette use has declined over time, consumption of other tobacco products has increased^12^. Although there is a strong cultural element in these trends, they may be in part explained by differences in tax structure and regulations between cigarette and non-cigarette products^12^. Most rigorous taxation policies applied to cigarettes have largely excluded other smoked tobacco products, such as cigars and cigarillos, loose tobacco for roll-your-own, and pipe tobacco^13^. Similarly, smokeless tobacco products, including snus, snuff and chewing tobacco have received limited attention in most countries, despite their increasing availability^14^.

Available evidence highlights the importance of price differentials between countries and across product types. The increase in the availability of non-cigarette products on the market further substantiates the need to understand price differentials beyond cigarettes. While a number of studies have examined prices and affordability of cigarette^6,15^ and non-cigarette tobacco products^16^, analyses on price differentials across a range of tobacco products and countries are lacking. The WHO provides country-level data on retail prices for cigarettes and a small number of non-cigarette products, and more recently on price dispersion (i.e. share of cheapest brand price in premium brand price) for cigarettes, however, the latter is not reported for non-cigarette products^1^. To address these gaps in the literature, we used commercial data to compare prices and price differentials of both cigarette and non-cigarette products across 79 countries worldwide.

## METHODS

### Data sources and measures

#### Tobacco price data

Retail price data for multiple tobacco products were obtained from Euromonitor Passport, an online database of the market research company Euromonitor International^17^. Euromonitor International annually collects market data from several retail sources, covering at least the ten brands with the highest market share within each country. Euromonitor has emerged as a useful data source in tobacco research^18-20^. Although Euromonitor International has recently started working with the tobacco industry, all data used in this analysis predate this collaboration^21^ .

We used retail price data collected in December 2016 covering 12 types of tobacco products – six smoking tobacco products and six smokeless tobacco products, across all countries for which data are reported. Smoking tobacco products and number of countries included were: cigarettes (79), cigarillos (48), cigars (56), fine-cut tobacco (38), heated tobacco (11), and pipe tobacco (32). Smokeless tobacco products and number of countries included were: Asian-style chewing tobacco (2), loose Swedish-style snus (2), portion Swedish-style snus (2), portion US-style moist snuff (1), US-style chewing tobacco (2), and other chewing tobacco (1).

Data collected included the pack size of the product, the unit of measurement (e.g. sticks, grams), price of each pack, price of one unit of product or unit price (e.g. one cigarette stick). In products recorded in grams (g) or ounces (fine-cut tobacco and smokeless tobacco products), prices were originally recorded per 1000 units, so these were divided by 1000 to obtain the price of a unit. Prices were recorded in local currencies in addition to US dollars (US$) converted by Euromonitor International. The prices of all tobacco products were made equivalent to a pack of 20 sticks of cigarettes for the purpose of comparison. This was done by equating either the product’s tobacco content or nicotine content to that of cigarettes, based on data available from previous studies. It was previously determined that one cigarette stick contained approximately 8.7 mg of nicotine and 0.7 g of tobacco^22,23^. We approximated one cigar stick as four cigarettes^24^, one cigarillo stick (3 g tobacco) as four cigarettes^25^, 25 g of pipe tobacco as 50 cigarettes^24^, 1 g of fine-cut tobacco as one cigarette^26^, and one heated tobacco stick (70% nicotine delivery relative to cigarettes) as 1.43 cigarettes^27,28^. For smokeless tobacco, 1 g of dry snuff (15.8 mg nicotine) was equivalent to about 1.82 cigarettes, 1 g of moist snuff (12.0 mg nicotine) was approximately equivalent to 1.38 cigarettes^29^, and 1 g of snus (10.46 mg nicotine) was approximately equivalent to 1.20 cigarettes^29^.

#### Country level data

Countries were categorized according to their geographical regions as defined by the WHO, namely African Region (AFRO), Eastern Mediterranean Region (EMRO), European Region (EURO), Region of the Americas (PAHO), South-East Asia Region (SEARO), and Western Pacific Region (WPRO)^30^.

#### Outcome measures

The main outcome of this study was price differentials of tobacco products. Price differentials were calculated for each product type in each country by expressing the minimum price as a percentage of the median. A higher percentage reflects a smaller gap between the minimum and median price, and vice versa. Median prices were calculated instead of the mean, given that the distribution of prices may be skewed, thereby mitigating the impact of outliers, and also in line with other studies that have examined price data from Euromonitor^18,20^.

### Statistical analysis

We considered each type of tobacco product with more than five price data points (i.e. product brands) collected within a country as valid data (273 data points were removed from 47 countries), with the exception of heated tobacco for which the small number of data points reflected the relatively lower availability of this product on the global market. Datasets were cleaned with a small number of implausible values removed.

Descriptive analyses were conducted using Excel. We calculated the median price per pack and price differentials for each of the 12 product types available in each country and, subsequently, present the mid-point and range of these outcome measures by their geographical regions. Price differentials of each product type for individual countries are additionally presented on world maps created using Stata version 15.0^30^.

## RESULTS

We analyzed 6920 price data points (i.e. product brands) for 12 tobacco product types across 79 countries, including: 5 countries in AFRO, 6 in EMRO, 39 in EURO, 15 in PAHO, 3 in SEARO, and 12 in WPRO (Supplementary file Table 1).

### Median prices of each product type

Median country-level cigarette prices ranged, across regions, from 2.00 US$ per pack in AFRO to 4.00 US$ in WPRO (Table 1). Generally, fine-cut tobacco and pipe tobacco were less expensive than cigarettes. Cigars were the most expensive smoking tobacco product across all regions. However, the large range in prices of cigars in the EMRO region suggests that there were less expensive options available with prices comparable to cigarettes. Prices of cigarillos varied considerably across regions (3.10 to 10.75 US$) and relative to cigarettes. Cigarillos were more expensive than cigarettes in most regions except for EURO and SEARO regions. Among the 12 countries with heated tobacco products, 10 were in the EURO region, with prices (median: 8.64 US$, range: 2.85–14.54) higher than cigarettes but within a wide range across EURO countries.

**Table 1:** Country-level median prices (US$ per 20-cigarette pack equivalency) of tobacco products by WHO geographical regions, 2016

Product type	AFRO^1^	EMRO^2^	EURO^3^	PAHO^4^	SEARO^5^	WPRO^6^
	Median (Range)	Median (Range)	Median (Range)	Median (Range)	Median (Range)	Median (Range)
**Smoking tobacco products**						
Cigarettes	2.00 (0.80–2.20)	2.60 (1.10–3.20)	3.80 (0.80–14.00)	3.60 (1.40–11.80)	2.40 (1.60–3.80)	4.00 (0.80–16.20)
Cigarillos	9.15*	7.65*	3.10 (0.57–18.85)	3.88 (2.55–10.62)	2.15 (0.97–15.15)	10.75 (6.65–29.5)
Cigars	29.96 (10.67–49.25)	43.60 (1.15–167.20)	25.48 (4.75–110.70)	35.04 (13.20–115.00)	59.50 (4.40–91.37)	82.09 (16.65–164.67)
Fine-cut tobacco	-	2.94*	1.85 (0.85–6.17)	1.21 (0.55–2.95)	1.13 (0.22–2.03)	4.90 (0.99–8.82)
Heated tobacco	-	4.40*	8.64 (2.85–14.54)	-	-	5.42*
Pipe tobacco	0.34 (0.27–0.40)	0.15 (0.10–0.21)	2.28 (0.81–7.88)	2.27 (1.44–3.32)	2.28*	5.63 (1.28–9.99)
**Smokeless tobacco products**						
Asian-style chewing tobacco	-	0.63*	-	-	0.76*	-
Loose Swedish-style snus	-	-	3.72 (2.17–5.27)	-	-	-
Other chewing tobacco	-	-	14.82*	-	-	-
Portion Swedish-style snus	-	-	5.83 (3.41–8.24)	-	-	-
Portion US-style moist snuff	0.26 (0.23–0.49)*	-	-	-	-	-
US-style chewing tobacco	-	-	48.48 (39.09–57.87)	-	-	-

1 AFRO: African Region (N=5) – Algeria, Cameroon, Kenya, Nigeria, South Africa.

2 EMRO: Eastern Mediterranean Region (N=6) – Egypt, Morocco, Pakistan, Saudi Arabia, Tunisia, United Arab Emirates.

3 EURO: European Region (N=39) – Austria, Azerbaijan, Belarus, Belgium, Bosnia-Herzegovina, Bulgaria, Croatia, Czech Republic, Denmark, Estonia, Finland, France, Georgia, Germany, Greece, Hungary, Ireland, Israel, Italy, Kazakhstan, Latvia, Lithuania, North Macedonia, Netherlands, Norway, Poland, Portugal, Romania, Russia, Serbia, Slovakia, Slovenia, Spain, Sweden, Switzerland, Turkey, Ukraine, United Kingdom, Uzbekistan.

>4 PAHO: Region of the Americas (N=15) – Argentina, Bolivia, Brazil, Canada, Chile, Colombia, Costa Rica, Dominican Republic, Ecuador, Guatemala, Mexico, Peru, Uruguay, USA, Venezuela.

5 SEARO: South-East Asia Region (N=3) – India, Indonesia, Thailand.

6 WPRO: Western Pacific Region (N=12) – Australia, Mainland China, Hong Kong, Japan, Malaysia, New Zealand, Philippines, Singapore, South Korea, Taiwan, Vietnam.

* Only one country.

Most smokeless tobacco products were observed in the EURO, and only one country in each of AFRO, EMRO, and SEARO. Among the four types of smokeless tobacco products in the EURO region, US-style chewing tobacco was the most expensive (48.48 US$) while loose Swedish-style snus was the least expensive (3.72 US$) (Table 1). EMRO and SEARO each had one country with Asian-style chewing tobacco products with comparable median prices (0.64 and 0.76 US$).

### Price differentials of each product type

**Figure 1:**
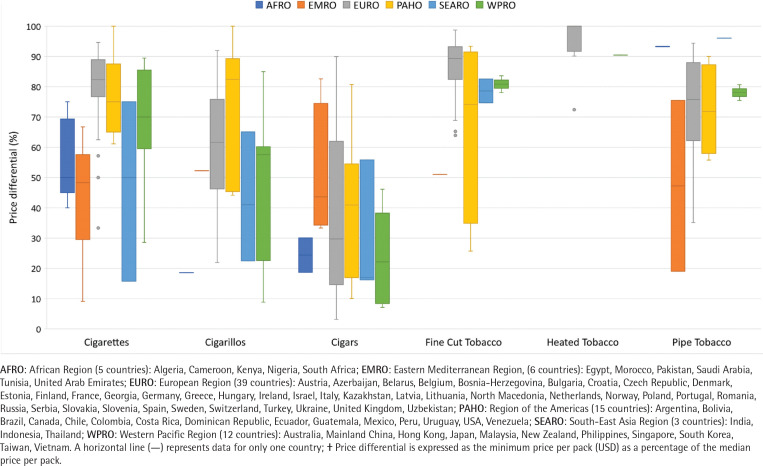
Country-level median price differentials**†** (%) of smoking tobacco products by WHO geographical regions, 2016

**Figure 2:**
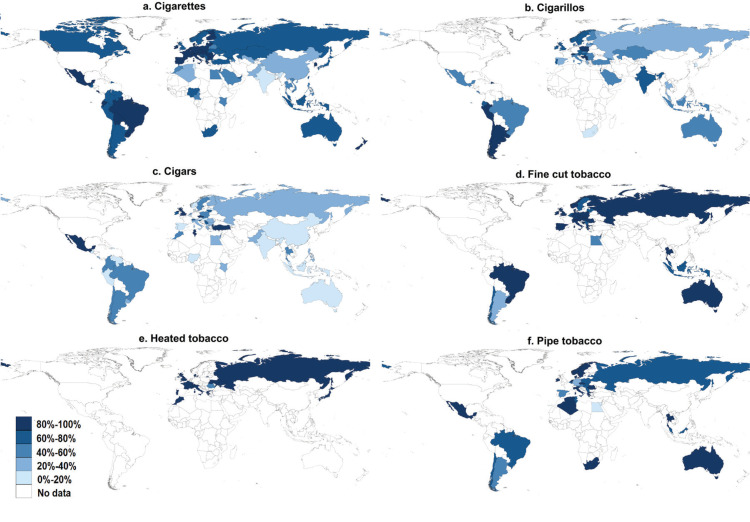
(a-f). Differential prices of smoking tobacco products in 79 countries in 2016, calculated as (Minimum price)/(Median price) × 100

The country-level median of price differentials varied across products and WHO regions (Figure 1 and Figure 2 a-f). Median price differentials of cigarettes were smaller in the EURO, PAHO and WPRO regions (minimum price ≥70% of median price) but larger in the EMRO, AFRO and SEARO regions (minimum price ≤50% of median price). Median price differentials of cigarillos ranged from 19% in the AFRO region to 82% in PAHO region. Heated tobacco products had the smallest price differentials among all smoking tobacco products (89–100%). With the exception of the EMRO region, price differentials of pipe tobacco and fine-cut tobacco were relatively small, but varied widely in the EURO and PAHO regions. The largest median price differentials across all smoking tobacco products and WHO regions were recorded for cigars (17–44%). Across smokeless tobacco products, price differentials were mostly small except for Asian-style chewing tobacco in the SEARO region. Supplementary file Table 2 provides the country-level median price differentials of tobacco products by WHO geographical region. Supplementary file Table 3 lists the price differentials of tobacco products by country.

## DISCUSSION

In this analysis of commercial data for 12 different types of tobacco products in 79 countries, we found an overall large variation in their median price and price differentials between countries and WHO regions.

Prices varied for both cigarettes and alternative tobacco products. Compared with other products, cigars were consistently the most expensive across all regions. By contrast, pipe tobacco and fine-cut tobacco were generally less expensive than cigarettes across all regions. Understanding of these cheaper alternatives of smoking tobacco to cigarettes is highly important as more price-sensitive users, such as adolescents and socioeconomically disadvantaged consumers, may potentially switch to cheaper priced products and maintain tobacco use^10^. Alarmingly, these alternative products are often less regulated than cigarettes. For instance, in the European Union (EU), taxation policies are more favorable for cigarillos than for cigarettes and they are not subject to the same product regulations as cigarettes (e.g. no restrictions on pack size, units sold per pack and characterizing flavours^31^). These disparities in taxation policies may also explain the relatively low cost of cigarillos in the EU^32^ and our findings that cigarillos were less expensive than cigarettes in the EURO region.

Regarding smokeless tobacco products, the price of Asian-style chewing tobacco was lower than cigarettes in the EMRO and SEARO region. Previous studies have indicated that despite recent efforts to increase the price of tobacco products in some SEARO countries, these policies have not adequately kept up with rapid economic growth, with smokeless tobacco products in particular found to be highly affordable in India^33^ and Bangladesh^34^. Furthermore, Asian-style chewing tobacco in the SEARO region had the largest price differential observed. Price differentials, which indicate the presence of a gap in prices between ‘premium’ and ‘budget’ products, can increase price minimization strategies such as switching to less expensive brands and subsequently undermine the effect of taxation policies^35^. Evidence supports that, like with cigarettes, increasing the price of non-cigarette products is also effective in reducing their consumption^16^. However, it has also been suggested that differential tax strategies for nicotine products based on relative health risk levels may encourage tobacco users of more harmful products to switch to less harmful ones^36^.

Taxation structure may account for observed price differentials across various product types and countries. Exploring how price differences within and across product types may be associated with different tax structures was beyond the scope of this analysis. However, evidence suggests that uniform and specific excise tax structure is most effective, while complex structures, such as *ad valorem* excise taxes, lead to greater variability in prices, which in turn can increase opportunities for switching to cheaper brands thus attenuating impact on smoking reduction^37^. For instance, following an *ad valorem* excise tax increase in 2009 in Thailand, a significant shift in consumption of upper price-tier cigarette brands towards lower price-tier brands was observed, thereby reducing the effectiveness of the taxation policy^38^. Future research can build on our findings and produce further evidence that will encourage governments to follow WHO guidance to implement specific or mixed excise systems with a minimum specific tax floor across the entire range of tobacco products^3^. Nevertheless, our findings do provide an overview of the types of tobacco products and geographical regions where price differentials are highest and therefore where research and policy attention are needed. For example, particular focus should be placed on lowering price differentials of cigarettes in EMRO, AFRO and SEARO regions, where very high levels were observed. Similarly, priority should be placed on lowering price differentials of Asian-style chewing tobacco in the SEARO region.

### Strengths and limitations

This study uses commercial data to examine prices and price differentials across several smoking and smokeless tobacco products in multiple countries covering all WHO regions. However, the cross-sectional design of the analysis provided a contemporary snapshot of the tobacco market worldwide but was unable to capture longer term changes that may impact the market and consumption. Although the data were available for 79 countries, the results may not reflect the situation in other countries. Nevertheless, we analyzed samples from a wide range of countries from all geographical regions.

In this analysis, we made some key assumptions about the best estimates for 1-unit equivalence of different tobacco products. However, their nicotine and tobacco content may not be uniform across countries^39^ and brands; similarly, the patterns of use, which influence the actual cost of tobacco to the consumer, may differ between products. For instance, while nicotine content may vary between products, tobacco users may compensate for lower content in the way they are used such as inhaling more frequently. Other studies have found different nicotine equivalencies, including differences across countries^39,40^. As such, price comparisons across different products should be interpreted with caution. Nevertheless, price differentials for the same product and country would not be impacted by equivalence estimates; although prices collected by Euromonitor may not capture the full range of prices in every single product and country, we are confident that the findings on price differentials within products largely reflect the actual variation in retail prices in 2016. To this end, while the data are a bit outdated, given that Euromonitor has since stopped publishing price data, this analysis is the first of its kind.

### Future research

While taxation and pricing fluctuate from time to time, this study usefully serves as a baseline for future research in the evaluation of the effectiveness of taxation and other policies in reducing tobacco price differentials. Although data were less robust for non-cigarette products, and sometimes only available in one country of a respective region for some product categories, it should be acknowledged that the WHO global reports on the tobacco epidemic only provide price differential data (using a different measure) for cigarettes. Thus, our findings highlight the importance of monitoring and reporting the prices and price differentials of other non-cigarette products where rigorous tobacco control policies are currently lacking. Future research should focus on analyzing data over time, and data sources should aim to capture more countries and products.

## CONCLUSIONS

This study offers a general global landscape of price differentials for smoking and smokeless tobacco products across multiple countries in all geographical regions. We found substantial variation between countries and regions across products, likely reflecting differences in taxation policies and structures. It is therefore critical to support countries in maximizing the impact of WHO FCTC Article 6 through implementing simple tobacco tax structures over complex ones, as a strategy to reduce price differentials. This study further highlights the gaps in taxation policies for non-cigarette tobacco products. Our findings can provide valuable insights for policy makers and highlight tobacco products and regions where tobacco prices require attention in order to minimize opportunities of switching to cheaper brands and products, and thus ensure that the population health benefits of taxation policies are fully realized.

## Data Availability

Data sharing is not applicable to this article as no new data were created.
