# Supplementary material for: Price differentials of tobacco products: A cross-sectional analysis of 79 countries from the six WHO regions
Source: Tob Induc Dis. 2021 Oct 15;19:80. doi: 10.18332/tid/142550 (PMC8519342; doi:10.18332/tid/142550)
Supplement: Supplementary file 1 [file TID-19-80-s1.pdf]

SUPPLEMENTARY TABLES

Supplementary Table 1. Count of products and countries by tobacco product type and WHO region

	AFRO[1]		EMRO[2]		EURO[3]		PAHO[4]		SEARO[5]		WPRO[6]		Overall	
	Products	*Countries*	*Products*	*Countries*	*Products*	*Countries*	*Products*	*Countries*	*Products*	*Countries*	*Products*	*Countries*	*Products*	*Countries*
Cigarettes	172	5	170	6	3321	38	381	15	182	3	591	11	4817	78
Cigarillos	9	1	11	1	351	29	76	9	24	3	54	5	525	48
Cigars	25	3	36	6	543	38	153	15	38	3	204	9	999	74
Heated Tobacco	-	-	3	1	26	10	-	-	-	-	13	1	42	12
Pipe Tobacco	24	4	13	3	237	39	33	7	10	2	44	4	361	59
Asian-Style Chewing Tobacco	-	-	5	1	-	-	-	-	31	1	-	-	36	2
Loose Swedish-Style Snus	-	-	-	-	21	2	-	-	-	-	-	-	21	2
Other Chewing Tobacco	-	-	-	-	15	2	1	1	-	-	-	-	16	3
Portion Swedish-Style Snus	-	-	-	-	71	2	3	2	-	-	-	-	74	4
Portion US-Style Moist Snuff	7	1	-	-	-	-	2	1	-	-	1	1	10	3
US-Style Chewing Tobacco	-	-	-	-	16	3	1	1	-	-	-	-	17	4

[1]AFRO: African Region (5 countries): Algeria, Cameroon, Kenya, Nigeria, South Africa
[2] EMRO: Eastern Mediterranean Region (6 countries): Egypt, Morocco, Pakistan, Saudi Arabia, Tunisia, United Arab Emirates
[3] EURO: European Region (39 countries): Austria, Azerbaijan, Belarus, Belgium, Bosnia-Herzegovina, Bulgaria, Croatia, Czech Republic, Denmark, Estonia, Finland, France, Georgia, Germany, Greece, Hungary, Ireland, Israel, Italy, Kazakhstan, Latvia, Lithuania, North Macedonia, Netherlands, Norway, Poland, Portugal, Romania, Russia, Serbia, Slovakia, Slovenia, Spain, Sweden, Switzerland, Turkey, Ukraine, United Kingdom, Uzbekistan
[4]PAHO: Region of the Americas (15 countries): Argentina, Bolivia, Brazil, Canada, Chile, Colombia, Costa Rica, Dominican Republic, Ecuador, Guatemala, Mexico, Peru, Uruguay, USA, Venezuela
[5]SEARO: South-East Asia Region (3 countries): India, Indonesia, Thailand
[6]WPRO: Western Pacific Region (12 countries): Australia, Mainland China, Hong Kong, Japan, Malaysia, New Zealand, Philippines, Singapore, South Korea, Taiwan, Vietnam

Supplementary Table 2. Country-level median price differentials† (%) of tobacco products by WHO geographical regions, 2016

Product type	**AFRO**[1] Price Differential (%) *Median (Range)*	**EMRO**[2] Price Differential (%) *Median (Range)*	**EURO**[3] Price Differential (%) *Median (Range)*	**PAHO**[4] Price Differential (%) *Median (Range)*	**SEARO**[5] Price Differential (%) *Median (Range)*	**WPRO**[6] Price Differential (%) *Median (Range)*
**Smoking tobacco products**						
Cigarettes	50.00 (40.00-70.00)	48.33 (9.09-66.67)	82.35 (33.33-94.59)	75.00 (61.11-100.00)	50.00 (15.79-75.00)	70.00 (28.57-89.47)
Cigarillos	18.58*	52.29*	61.62 (21.95-91.93)	82.43 (44.16-100.00)	41.03 (22.44-65.12)	57.59 (8.84-84.96)
Cigars	24.39 (18.73-30.05)	43.6 (33.33-82.61)	29.74 (3.11-89.90)	40.9 (10.07-80.70)	17.05 (16.21-55.81)	22.15 (7.12-46.15)
Fine Cut Tobacco	-	51.02*	89.29 (63.91-98.74)	74.12 (25.67-93.33)	78.60 (74.68-82.52)	80.83 (83.62-78.05)
Heated Tobacco	-	100.00*	100.00 (56.83-100.00)	-	-	89.47*
Pipe Tobacco	93.27 (92.22-93.32)	47.26 (19.03-75.48)	75.76 (35.16-94.32)	71.83 (55.77-90.00)	96.03*	78.05 (75.47-80.62)
**Smokeless tobacco products**						
Asian-Style Chewing Tobacco	-	100.00*	-	-	16.67*	-
Loose Swedish-Style Snus	-	-	76.11 (63.75-88.47)	-	-	-
Other Chewing Tobacco	-	-	100.00*	-	-	-
Portion Swedish-Style Snus	-	-	68.59 (47.18-90.00)	-	-	-
Portion US-Style Moist Snuff	85.71*	-	-	-	-	-
US-Style Chewing Tobacco	-	-	99.55 (99.10-100.00)	-	-	-

[1]AFRO: African Region (5 countries): Algeria, Cameroon, Kenya, Nigeria, South Africa
[2] EMRO: Eastern Mediterranean Region (6 countries): Egypt, Morocco, Pakistan, Saudi Arabia, Tunisia, United Arab Emirates
[3] EURO: European Region (39 countries): Austria, Azerbaijan, Belarus, Belgium, Bosnia-Herzegovina, Bulgaria, Croatia, Czech Republic, Denmark, Estonia, Finland, France, Georgia, Germany, Greece, Hungary, Ireland, Israel, Italy, Kazakhstan, Latvia, Lithuania, North Macedonia, Netherlands, Norway, Poland, Portugal, Romania, Russia, Serbia, Slovakia, Slovenia, Spain, Sweden, Switzerland, Turkey, Ukraine, United Kingdom, Uzbekistan
[4]PAHO: Region of the Americas (15 countries): Argentina, Bolivia, Brazil, Canada, Chile, Colombia, Costa Rica, Dominican Republic, Ecuador, Guatemala, Mexico, Peru, Uruguay, USA, Venezuela
[5]SEARO: South-East Asia Region (3 countries): India, Indonesia, Thailand
[6]WPRO: Western Pacific Region (12 countries): Australia, Mainland China, Hong Kong, Japan, Malaysia, New Zealand, Philippines, Singapore, South Korea, Taiwan, Vietnam

*N= 1 country † Price differential is expressed as the minimum price per pack (USD) as a percentage of the median price per pack

Supplementary Table 3. Median price differentials† (%) of tobacco products by country, 2016

Country	*WHO Region*	Cigarettes	Cigarillos	Cigars	Fine Cut Tobacco	Heated Tobacco	Pipe Tobacco	Loose Swedish-Style Snus	Asian-Style Chewing Tobacco	Other Chewing Tobacco	Portion Swedish-Style Snus	Portion US-Style Moist Snuff	US-Style Chewing Tobacco
**Algeria**	AFRO	40.00					93.32					85.71	
**Argentina**	PAHO	64.71	82.43	42.83	25.67		55.77						
**Australia**	WPRO	73.08	57.59	8.28	83.62		80.62						
**Austria**	EURO	92.00	70.00	13.28	92.65		57.76						
**Azerbaijan**	EURO	57.14											
**Belarus**	EURO	50.00					67.90						
**Belgium**	EURO	81.25		15.93	68.87		69.56						
**Bolivia**	PAHO	81.82											
**Bosnia-Herzegovina**	EURO	83.33	91.94		86.67								
**Brazil**	PAHO	100.00	45.61	56.77	85.79		78.97						
**Bulgaria**	EURO	92.86	52.21	20.20									
**Cameroon**	AFRO	50.00											
**Canada**	PAHO	72.73											
**Chile**	PAHO	75.00	85.16	57.17	62.44		64.70						
**China**	WPRO	28.57		7.13									
**Colombia**	PAHO	71.43		47.64									
**Costa Rica**	PAHO	87.50	44.16	38.97									
**Croatia**	EURO	62.50											
**Czech Republic**	EURO	94.12	71.07	84.40	83.44		92.12						
**Denmark**	EURO	86.67	48.89	7.02	84.79	100.00	88.31			100.00			100.00
**Dominican Republic**	PAHO	65.00	100.00	10.07									
**Ecuador**	PAHO	86.21	61.18	43.48									
**Egypt**	EMRO	54.55		33.33	51.02		19.03						
**Estonia**	EURO	78.95			90.62								
**Finland**	EURO	93.75	53.42	31.45	89.61								
**France**	EURO	94.59				100.00							
**Georgia**	EURO	71.43	58.33	11.32									
**Germany**	EURO	80.65			93.83		35.16						
**Greece**	EURO	81.82	37.29	35.56	97.14	100.00	92.52						
**Guatemala**	PAHO	64.29		16.96									
**Hong Kong, China**	WPRO	86.49	84.96	39.11									
**Hungary**	EURO	89.47	69.57	3.11	84.46		75.76						

Country	*WHO Region*	Cigarettes	Cigarillos	Cigars	Fine Cut Tobacco	Heated Tobacco	Pipe Tobacco	Loose Swedish-Style Snus	Asian-Style Chewing Tobacco	Other Chewing Tobacco	Portion Swedish-Style Snus	Portion US-Style Moist Snuff	US-Style Chewing Tobacco
**India**	SEARO	15.79	65.12	16.22					16.67				
**Indonesia**	SEARO	75.00	41.03	17.05	74.68								
**Ireland**	EURO	80.33	77.65	62.56	89.25		94.32						
**Israel**	EURO	88.61	76.84	55.35	90.05								
**Italy**	EURO	88.46	59.46	29.74	98.74	100.00	81.25						
**Japan**	WPRO	61.90				90.48							
**Kazakhstan**	EURO	80.00	50.82										
**Kenya**	AFRO	50.00		30.05									
**Latvia**	EURO	88.24	46.15	6.96	63.91		76.77						
**Lithuania**	EURO	82.35	26.37	67.37	77.69								
**North Macedonia**	EURO	71.43	90.76	16.28	89.29		71.67						
**Malaysia**	WPRO	70.00	22.63	8.56	78.05		75.48						
**Mexico**	PAHO	81.82	45.10	80.70			90.00						
**Morocco**	EMRO	46.67		50.00									
**Netherlands**	EURO	88.89		24.90	65.22		87.38						
**New Zealand**	WPRO	86.42											
**Nigeria**	AFRO	75.00		18.74									
**Norway**	EURO	77.14	74.83	11.29	94.21		87.61	88.47			90.00		99.10
**Pakistan**	EMRO	36.36		37.20					100.00				
**Peru**	PAHO	61.11	93.28	12.96									
**Philippines**	WPRO	57.14		35.74									
**Poland**	EURO	94.12	81.69	56.08	84.21		60.78						
**Portugal**	EURO	88.00	62.96	19.65	96.62	100.00							
**Romania**	EURO	85.00	46.27	29.13	90.30	72.41	83.25						
**Russia**	EURO	71.43	21.95	25.41	95.00	100.00	72.03						
**Saudi Arabia**	EMRO	50.00	52.29										
**Serbia**	EURO	80.00	84.48	72.22	69.23		62.22						
**Singapore**	WPRO	84.62	60.17	46.15									
**Slovakia**	EURO	87.50	42.71	51.88	88.78		62.20						
**Slovenia**	EURO	89.47	61.62	78.57	90.67								
**South Africa**	AFRO	63.64	18.58				93.22						
**South Korea**	WPRO	89.47	8.84										
**Spain**	EURO	88.00	29.17	6.53	97.06		51.74						

Country	*WHO Region*	**Cigarettes**	**Cigarillos**	**Cigars**	**Fine Cut Tobacco**	**Heated Tobacco**	**Pipe Tobacco**	**Loose Swedish-Style Snus**	**Asian-Style Chewing Tobacco**	**Other Chewing Tobacco**	**Portion Swedish-Style Snus**	**Portion US-Style Moist Snuff**	**US-Style Chewing Tobacco**
**Sweden**	EURO	80.00	64.06	40.12	68.84		93.96	63.75			47.18		
**Switzerland**	EURO	89.41	87.50	61.48	89.29	90.14							
**Taiwan**	WPRO	63.64											
**Thailand**	SEARO	50.00	22.44	55.81	82.52		96.03						
**Tunisia**	EMRO	9.09		82.61			75.48						
**Turkey**	EURO	75.00	44.30	89.90									
**Ukraine**	EURO	75.00				100.00							
**United Arab Emirates**	EURO	66.67											
**United Kingdom**	EURO	76.67	71.72	85.94	81.29	92.16							
**Uruguay**	PAHO	90.48	83.50	30.29	93.33								
**USA**	EURO	89.83											
**Uzbekistan**	EURO	33.33											
**Venezuela**	PAHO	65.00		17.05									
**Vietnam**	WPRO	50.00											

† Price differential is expressed as the minimum price per pack (USD) as a percentage of the median price per pack

© 2021 Kyriakos C.N. et al.
